# Hemin with Peroxidase Activity Can Inhibit the Oxidative Damage Induced by Ultraviolet A

**DOI:** 10.3390/cimb44060183

**Published:** 2022-06-10

**Authors:** Wenli Hui, Zhipeng Yang, Ke Fang, Mengdi Wu, Wenhua Mu, Cong Zhao, Dan Xue, Tengteng Zhu, Xiao Li, Ming Gao, Yunhua Lu, Kunping Yan

**Affiliations:** College of Life Science, Northwest University, No. 229 Taibai Bei Road, Xi’an 710069, China; huiwl@nwu.edu.cn (W.H.); 18995328587@163.com (Z.Y.); 15229548269@163.com (K.F.); wmd@peihua.edu.cn (M.W.); mwh15829384334@163.com (W.M.); zhao15353957953@163.com (C.Z.); xuedan2020226@163.com (D.X.); zhutengt417@163.com (T.Z.); lx13253379340@163.com (X.L.); gm1477254506@163.com (M.G.); l572066129@163.com (Y.L.)

**Keywords:** UVA, HaCaT, hemin, hydrogen peroxide, tyrosine

## Abstract

Excessive reactive oxygen species (ROS), a highly reactive substance that contains oxygen, induced by ultraviolet A (UVA) cause oxidative damage to skin. We confirmed that hemin can catalyze the reaction of tyrosine (Tyr) and hydrogen peroxide (H_2_O_2_). Catalysis was found to effectively reduce or eliminate oxidative damage to cells induced by H_2_O_2_ or UVA. The scavenging effects of hemin for other free-radical ROS were also evaluated through pyrogallol autoxidation, 1,1-diphenyl-2-picrylhydrazyl radical (DPPH·)-scavenging assays, and phenanthroline–Fe^2+^ assays. The results show that a mixture of hemin and tyrosine exhibits strong scavenging activities for H_2_O_2_, superoxide anion (O_2_^−^·), DPPH·, and the hydroxyl radical (·OH). Furthermore, the inhibition of oxidative damage to human skin keratinocyte (HaCaT) cells induced by H_2_O_2_ or UVA was evaluated. The results show that catalysis can significantly reduce the ratio of cell apoptosis and death and inhibit the release of lactate dehydrogenase (LDH), as well as accumulation of malondialdehyde (MDA). Furthermore, the resistance to apoptosis was found to be enhanced. These results show that the mixture of hemin and tyrosine has a significantly protective effect against oxidative damage to HaCaT cells caused by UVA, suggesting it as a protective agent for combating UVA damage.

## 1. Introduction

Ultraviolet rays cause skin damage by inducing oxidative stress (OS) and prime photo aging and cancer in skin cells [1,2,3]. Ultraviolet A (UVA) constitutes 90% of ultraviolet light and can penetrate keratinocytes and eventually cause cell apoptosis [4]. The response induced by UVA irradiation is complex but primarily mediated by the formation of intracellular reactive oxygen species (ROS) [5], which oxidize unsaturated fatty acids in the membrane and induce a series of reactions involving lipid oxidation products that damage other biological macromolecules [6]. UVA also causes DNA damage, which includes the oxidation of the ribose and the base breakage of DNA strands, increasing the probability of cancer [7,8,9]. In addition, apoptosis, which is also involved in the pathogenesis of various skin diseases, is increased by UVA [10].

Hemin, a porphyrin ring compound containing iron, is widely used in food and medicine. Hemin is an anti-inflammatory agent with antioxidant effects. It improves anemia, suppresses pulmonary hypertension [11,12,13,14], and accelerates wound healing in diabetic rats [15]. In a previous study, we observed peroxidase activity of ferrous hemoglobin that catalyzed the reaction of hydrogen peroxide with tyrosine. We also observed that it reduced the oxidative damage of human umbilical vein endothelial cells induced by hydrogen peroxide and verified that the heme was the catalytic center of the ferrous hemoglobin [16]. Furthermore, we wanted to explore whether the heme exhibited catalytic activity when it was released from the three-dimensional structure of hemoglobin.

With the present study, we aimed to investigate the peroxidase effect of hemin—the main form of heme—and evaluate the free-radical-scavenging ability of hemin and, thus, its protective effect on oxidative damage caused by hydrogen peroxide or UVA in vitro, including reducing lipid peroxidation, cell-cycle arrest, and apoptosis.

## 2. Materials and Methods

### 2.1. Materials

Hemin was purchased from Kemin (Shanghai, China). Tyrosine, glucose oxidase (GOX), vitamin C (VC), and vitamin E (VE) were purchased from Sigma (St. Louis, MO, USA). Pyrogallol was purchased from Comeo (Tianjin, China). Phenanthroline was purchased from Dengfeng (Tianjin, China). Butylated hydroxytoluene (BHT) was purchased from J&K (Beijing, China).

### 2.2. Measurement of Fluorescence

Fluorescence data were obtained using an F4500 fluorescence spectrophotometer (Hitachi, Japan). The dityrosine was measured at EX: 320 nm and EM: 411 nm at 25 °C, with the slit set to 10 nm, a PMT voltage of 700 V, and a scanning speed of 120 nm/min.

### 2.3. Antioxidant Activity Assay

#### 2.3.1. Superoxide Anion Free-Radical-Scavenging Assay

The scavenging activity of hemin against superoxide anion free radicals (O_2_^−^·) was measured by inhibiting the efficiency of pyrogallol autoxidation at 325 nm with an SPD-10AVP Plus UV-vis spectrophotometer (Shimadzu, Japan) and calculated using the following formula [17,18].

(1) $$SA\left(\%\right)=\frac{A_{s}-A_{0}}{A_{0}}\times 100\%\;$$

where *SA* is the scavenging activity of the sample to be tested, *A_s_* is its absorbance, and *A*_0_ is the absorbance of the sample blank.

#### 2.3.2. DPPH· Scavenging Assay

DPPH· is a stable nitrogen free radical; the decrease in the absorbance of DPPH· is proportional to the free-radical-scavenging rate. According to previous reports, the scavenging activity of hemin for DPPH· was measured at 517 nm and calculated using the following formula [19,20].

(2) $$SA\left(\%\right)=1- [\frac{A_{s}-A_{0}}{A_{c}}]\times 100\%\;$$

where *SA* is the scavenging activity of the sample, *A_s_* is the absorbance of the DPPH· and the sample, *A*_0_ is the absorbance of the solvent of the sample and methanol, and *Ac* is the absorbance of the DPPH· and solvent of the sample.

#### 2.3.3. Hydroxyl Radical Scavenging

The hydroxyl radical (·OH) was produced through a Fenton reaction, and the scavenging activity was measured using the iron complex of phenanthroline at 536 nm and calculated according to the following formula [21].

(3) $$SA\left(\%\right)=\frac{A_{s}-A_{c}-A_{1}}{A_{0}-A_{1}}\times 100\%\;$$

where *SA* is the scavenging activity of the sample, *A_s_* is the absorbance of the sample, *A_c_* is the absorbance of the tube without adding H_2_O_2_, *A*_1_ is the absorbance of the tube without adding the sample, and *A*_0_ is the absorbance of the tube without adding H_2_O_2_ and the sample.

### 2.4. Cell Culture and UVA Treatment

The HaCaT cells in this study were from the ATCC (American Type Culture Collection) and were cultured in RPMI 1640 medium with 10% fetal bovine serum and penicillin/streptomycin (1%, Sigma) at 37 °C in a humidified atmosphere of 95% air and 5% CO_2_.

Cells were treated with GOX, Tyr-GOX, Hemin-GOX, Hemin-Tyr-GOX, VC-GOX, VE-GOX, and BHT-GOX and incubated at 37 °C under 5% CO_2_. For the MTT and LDH assays, the cells (200 μL/hole) were subcultured in 96-well cell culture plates. For measurements of MDA, cell-cycle arrest, and cell apoptosis, the cells (2 mL/hole) were subcultured in a 6-well cell culture plate. Control-group cells were not incubated with GOX but were treated with PBS.

For UVA-induced oxidative damage, the drugs (hemin, Tyr, VC, VE, and BHT) and concentrations were the same as those used for GOX-induced oxidative damage in cells. For the MTT and LDH assays, the medium was discarded after the cells (200 μL/hole) were grown in the 96-well cell culture plate for 24 h; then, the cells were washed with PBS, and 50 μL of drugs was added to each well. Then, the HaCaT cells were exposed to UVA radiation at 30 J/cm^2^. Finally, 150 μL of drugs was added to each well for subsequent assays. For measurement of MDA, cell-cycle arrest, and cell apoptosis, the treatment methods were similar to those mentioned above, but 500 μL of drugs was added before irradiation, and 1.5 mL of drugs was added after irradiation. Control-group cells were not irradiated with UVA and treated with PBS.

### 2.5. Cell Viability Assay

The cells were incubated for 24 h, and 3-(45)-dimethylthiahiazol (-z-y1)-35-di-phenytetrazoliumromide (MTT) was added to each well after drug treatment for 24 h. After incubation for 4 h, the supernatant was removed. The formazan crystals in each well were dissolved in dimethyl sulfoxide (DMSO), and the absorbance at 490 nm was measured on a scanning multi-well spectrophotometer (BioTek, Winooski, VT, USA).

### 2.6. Measurement of LDH and MDA Release

LDH and MDA were assessed with an LDH cytotoxicity assay kit (Beyotime, Shanghai, China) and a lipid peroxidation MDA assay kit (Beyotime, Shanghai, China), respectively, according to the manufacturers’ instructions.

### 2.7. Annexin-V–Propidium Iodide Assay

All of the cells were harvested and washed with PBS and resuspended in annexin V–FITC binding buffer. Annexin V–fluorescein isothiocyanate and PI were added to this cell suspension. Then, the cells were incubated for 15 min and analyzed by flow cytometry (Beckman Coulter-Elite, Fullerton, CA, USA). The data were analyzed with Expo32 V1.2 analysis software (Beckman Coulter-Elite, Brea, CA, USA).

### 2.8. Cell-Cycle Analysis

The cells were trypsinized and centrifuged for 10 min. The fresh pellets were washed with PBS, fixed with ethanol, and incubated overnight. The remaining ethanol was then removed, and the cells were resuspended in PBS containing RNase A and PI. The resulting mixture was incubated for 30 min and immediately analyzed by flow cytometry. The proportions of cells in the phases were quantified with ModFit 3.2 software (Verity Software House, Topsham, ME, USA).

### 2.9. Statistical Analysis

All the experiments were independently performed at least 3 times, and the results were expressed as the means ± SDs. GraphPad Prism 8.0 software (San Diego, CA, USA) was used for statistical analysis. The results were subjected to one-way ANOVA, and *p* < 0.05 was regarded as statistically significant.

## 3. Results

### 3.1. Catalysis of Hemin

**Figure 1:**
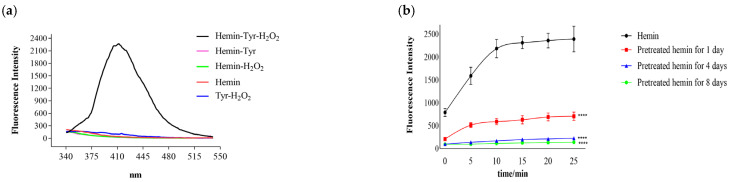
Hemin catalyzes the reaction of tyrosine and hydrogen peroxide to produce dityrosine with fluorescence. (**a**) The addition of hemin increased the fluorescence intensity of the mixture of tyrosine and hydrogen peroxide. Reaction mixtures were prepared containing 0.7 μM hemin, 0.1 mM Tyr, and 1 mM H_2_O_2_. (**b**) Catalysis of the hemin pretreated with hydrogen peroxide or not: the reaction time was 25 min, the fluorescence intensity was detected every 5 min, the results are expressed as the means ± SDs (*n* = 8), and the last data points show comparisons with hemin at 25 min; **** *p* < 0.0001.

Tyrosine is oxidized by hydrogen peroxide to dityrosine, which has a strong fluorescence property (EXmax: 320 nm; EMmax: 411 nm) [22]. As shown in Figure 1a, the fluorescence intensity produced by the mixture of tyrosine and hydrogen peroxide or tyrosine alone was low; however, it was significantly increased after adding hemin to the mixture of tyrosine and hydrogen peroxide and higher than all the controls, which suggests that hemin catalyzed the reaction of tyrosine and hydrogen peroxide. In this reaction, hemin can be oxidized gradually by hydrogen peroxide [23], and its oxidation products probably catalyze the reaction. In order to rule out this possibility, the hemin was first pretreated with excess hydrogen peroxide (100 mM) to produce more oxidation products. Additionally, the reaction of tyrosine and hydrogen peroxide was then catalyzed by the pretreated hemin. As shown in Figure 1b, the catalysis gradually decreased with hemin pretreatment time, whereas the fluorescence intensity produced by the catalysis was much lower than that for the unpretreated hemin. The oxidation and degradation of hemin were slow processes. Additionally, the accumulation of hemin oxidation products did not enhance the catalysis; instead, the fluorescence intensity decreased with the degradation of hemin. This suggests that the catalysis was caused by the hemin itself rather than its oxidation products.

### 3.2. Effects of Hemin and Substrate Concentration on the Catalytic Reaction

**Figure 2:**
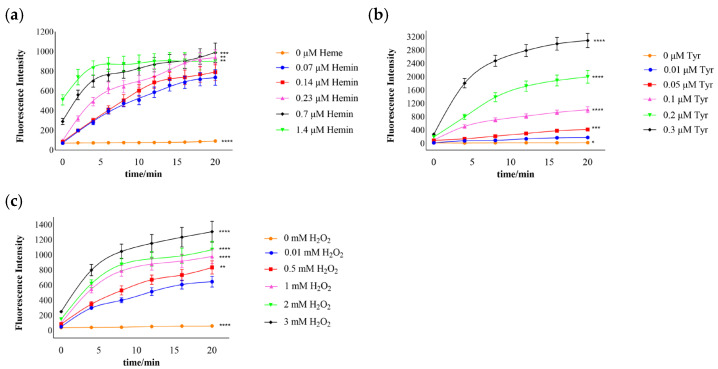
Catalysis was enhanced with increases in the catalyst and substrate concentrations. (**a**) Enhancement of catalysis with the hemin, Tyr at 0.1 mM, and H_2_O_2_ at 1 mM; the last data points show comparisons with 0.07 μM hemin at 20 min. (**b**) Catalysis enhancement with the tyrosine, hemin at 0.7 μM, and H_2_O_2_ at 1 mM; the last data points show comparisons with 0.01 μM Tyr at 20 min. (**c**) Catalysis enhancement with the hydrogen peroxide, hemin at 0.7 μM, and Tyr at 0.1 mM; the last data points show comparisons with 0.01 mM H_2_O_2_ at 20 min. The results are expressed as the means ± SDs (*n* = 8); * *p* < 0.05, ** *p* < 0.01, *** *p* < 0.001, and **** *p* < 0.0001.

The production of dityrosine increased with time and hemin, which indicates that the catalytic effect was enhanced with increased hemin (Figure 2a). When the volume of hemin was 1.4 μM, its catalysis was not increased, which probably indicates that the catalysis of hemin was limited at this substrate concentration. As shown in Figure 2b, with increased tyrosine and time, dityrosine also increased and reached a peak at 0.3 μM tyrosine and 20 min. An increase in dityrosine with time and H_2_O_2_ was noted (Figure 2c). These results suggest that the catalytic activity increased with the catalyst and substrate concentrations.

### 3.3. Free-Radical Scavenging by Hemin

Oxidative damage can be induced by excessive free radicals in the body. We also evaluated the scavenging activity of superoxide anion free radicals, DPPH·, and hydroxyl free radicals of hemin, tyrosine, and their mixture in vitro. Furthermore, we explored the effects of increases in the hemin and tyrosine concentrations in the mixture (hemin–Tyr) on the scavenging activity.

#### 3.3.1. Superoxide Anion Free-Radical Scavenging

**Figure 3:**
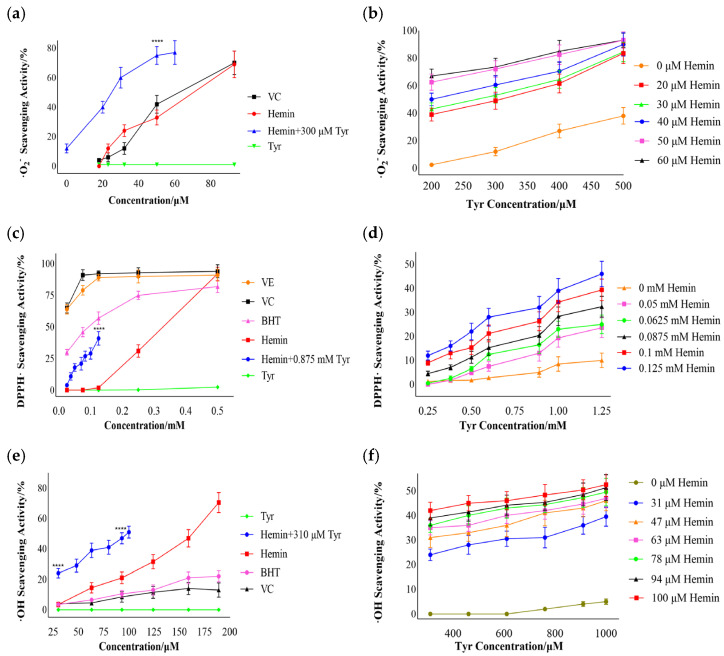
The mixture of hemin and tyrosine exhibited radical-scavenging activities. (**a**) The superoxide anion-scavenging activity of the mixture (hemin-Tyr) with added tyrosine was 300 µM; the data points show comparisons with 50 μM hemin, **** *p* < 0.0001. (**b**) Effects of tyrosine and hemin on superoxide anion-scavenging ability for the mixture (hemin-Tyr). (**c**) DPPH·-scavenging activity of the mixture (hemin-Tyr) with added tyrosine was 0.875 mM; the data points show comparisons with 0.125 mM hemin, **** *p* < 0.0001. (**d**) Effects of tyrosine and hemin on the DPPH·-scavenging ability of the mixture (hemin-Tyr). (**e**) Hydroxyl free-radical-scavenging activity of the mixture (hemin-Tyr) with added tyrosine was 310 µM; the dots show comparisons with 30 and 90 μM hemin, **** *p* < 0.0001. (**f**) Effects of tyrosine and hemin on the hydroxyl free-radical-scavenging ability of the mixture. Results are expressed as means ± SDs (*n* = 6).

Based on Figure 3a, it is evident that in the range of 20-80 μM, tyrosine had a minimal scavenging effect on the superoxide anion, whereas hemin and vitamin C were similar. Additionally, the addition of hemin significantly enhanced the scavenging effect of tyrosine. The scavenging activities of 300 μM tyrosine and 50 μM hemin were 12% and 33%, respectively; however, the activity reached 75% after mixing them. This indicates that the superoxide anion-scavenging activity of the mixture of hemin and tyrosine was significantly increased relative to that of hemin or tyrosine alone; it was not a simple sum of their respective scavenging effects but a synergistic enhancement effect. Figure 3b shows that tyrosine has a scavenging capacity of more than 200 μM; when the hemin was unchanged, the scavenging effect of the mixture was enhanced with an increase in tyrosine. These results indicate that the mixture of hemin and tyrosine exhibits significant scavenging activity for superoxide anion free radicals. Unfortunately, the limitations of solubility imposed by the different reaction system prevented the analysis of the mixture’s scavenging effect at higher concentrations.

#### 3.3.2. DPPH·Scavenging

As shown in Figure 3c, the scavenging ability of hemin was weaker than that of vitamin C, vitamin E, or BHT at low concentrations (0–0.45 mM). When the concentration of hemin was above 0.45 mM, the scavenging effect was similar to that of the positive control. The scavenging activity of 0.125 mM hemin and 0.875 mM tyrosine was 2% and 5%, respectively; however, the activity increased to 41% when they were mixed. This indicates that hemin and tyrosine also had a synergistic enhancement effect on DPPH scavenging. As shown in Figure 3d, tyrosine alone had a low scavenging ability for DPPH, and the scavenging ability of the mixture was also gradually enhanced with an increase in tyrosine or hemin.

#### 3.3.3. Hydroxyl Free-Radical Scavenging

Figure 3e shows that hemin had a significant scavenging ability for hydroxyl free radicals compared to vitamin C and BHT; when the concentration of hemin was 190 μM, the scavenging ability was 71%. A tyrosine concentration of 310 μM had no scavenging effect for hydroxyl free radicals; however, the scavenging effect of hemin mixed with 310 μM tyrosine was 20–26% higher than that of hemin alone in the range of 30–100 μM. As shown in Figure 3f, tyrosine had a poor scavenging effect on hydroxyl free radicals; the scavenging rate was 5% for concentrations up to 1 mM, and the scavenging ability of the mixture was also gradually enhanced with an increase in tyrosine or hemin. These results indicate that the mixture of hemin and tyrosine has a synergistic scavenging effect on hydroxyl free radicals.

### 3.4. Protection by Hemin Catalysis against the Oxidative Damage of Cells Induced by GOX

With a short lifespan of about 1 ns, the superoxide anion is transformed to stable hydrogen peroxide by the action of superoxide dismutase in cells. Additionally, hydrogen peroxide produces strongly oxidizing hydroxyl free radicals, which damage cells through a Fenton reaction [24]; hence, its removal is of great significance for resisting oxidative damage. The protective effects of hemin and tyrosine against the HaCaT damage induced by hydrogen peroxide was explored.

**Figure 4:**
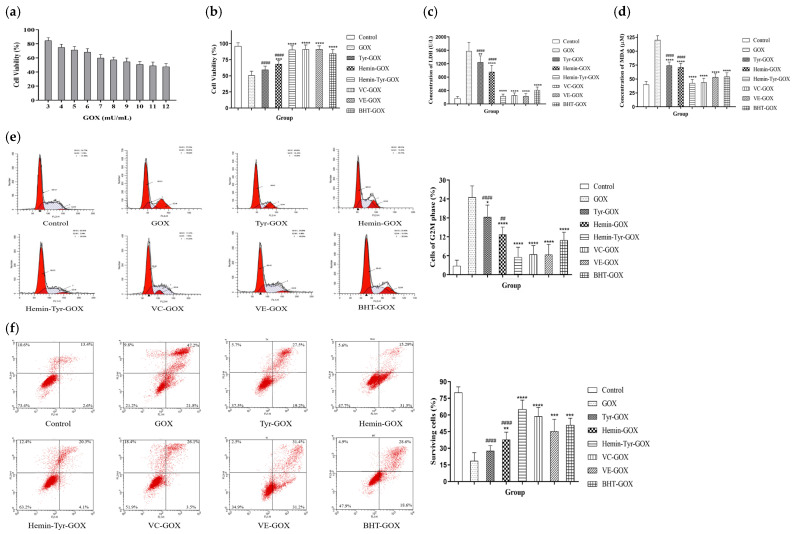
The mixture of hemin and tyrosine protected HaCaT cells from GOX-induced oxidative damage. (**a**) The half-maximal inhibitory concentration of GOX was 10 mU/mL; after adding GOX, HaCaTs were incubated with FBS-free medium and a different medium containing 50 μM hemin, 200 μM tyrosine, 50 μM vitamin C, 50 μM vitamin E, or 50 μM BHT. (**b**) Then, the cell viability of (**c**) LDH and (**d**) MDA was measured. (**e**) Cell cycle and (**f**) apoptosis detected by flow cytometry. Data are shown as the means ± SDs (*n* = 6), compared with the GOX group; * *p* < 0.05, ** *p* < 0.01, *** *p* < 0.001, and **** *p* < 0.0001, compared with the hemin-Tyr-GOX group;, ^##^ *p* < 0.01, and ^####^ *p* < 0.0001.

Glucose oxidase (GOX) specifically catalyzes the conversion of β-D-glucose into gluconic acid and hydrogen peroxide and maintains the stability of hydrogen peroxide concentration. Figure 4a shows that the concentration of GOX corresponding to the half-maximal inhibitory concentration was 10 mU/mL. According to Figure 4b, the cell viability of the mixture group (hemin–Tyr–GOX) was 90%, which was significantly higher than that of the GOX-alone treatment group, at 50%. The viability in the mixture group was much higher than that for hemin or tyrosine alone (hemin: 68%; Tyr: 59%). Furthermore, the degree of cell damage was evaluated according to the concentration of LDH released after the plasma membrane was broken. Figure 4c shows that the activity of LDH in the hemin–tyrosine mixture treatment group was 229 U/L, which is significantly lower than the 1578 U/L in the GOX-alone treatment group, and the extravasation of LDH from cells was strongly inhibited. Given that the MDA level reflects the level of lipid peroxidation in cells, as shown in Figure 4d, the MDA (120 μM) in the GOX-alone treatment group was significantly higher than that in the control group (40 μM), while the mixture group (42 μM) showed a result close to that of the control group. In addition, both the LDH release and MDA accumulation for the hemin–tyrosine mixture treatment were significantly lower than those for the single treatment, demonstrating a synergistic scavenging action for hemin and tyrosine for hydrogen peroxide (Figure 4c,d).

Excessive hydrogen peroxide is also a potential cause of cell-cycle arrest and apoptosis. As shown in Figure 4e, 24% of the cells treated with GOX alone were blocked in the G2–M phase, whereas only 5% treated with the mixture were blocked. After GOX treatment, the cells continuously produced H_2_O_2_, significantly contributing to cell apoptosis, with a survival rate of only 18%. However, the number of viable cells in the group treated with the hemin–tyrosine mixture reached 65%, which is higher than that of the other treatment groups, except for the control group, which had a survival rate of 80% (Figure 4f). These results indicate that the mixture of hemin and tyrosine prevented the occurrence of cell-cycle arrest and apoptosis induced by hydrogen peroxide.

### 3.5. Effects of Hemin and Tyrosine on UVA-Induced Photodamage in HaCaT Cells

**Figure 5:**
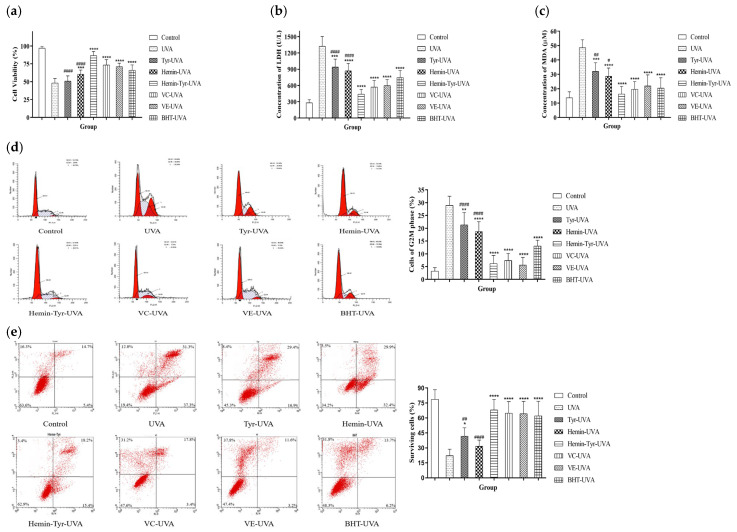
The mixture of hemin and tyrosine protected HaCaT cells from UVA-induced oxidative damage. After UVA irradiation, HaCaTs were incubated with an FBS-free medium and a different medium containing 50 μM hemin, 200 μM tyrosine, 50 μM vitamin C, 50 μM vitamin E, or 50 μM BHT. (**a**) Then, the cell viability of (**b**) LDH and (**c**) MDA was measured by MTT assay. (**d**) Cell-cycle and (**e**) apoptosis detected by flow cytometry. Data are shown as the means ± SDs (*n* = 6), compared with the UVA group; * *p* < 0.05, ** *p* < 0.01, *** *p* < 0.001, and **** *p* < 0.0001, compared with the hemin-Tyr-UVA group; ^#^ *p* < 0.05, ^##^ *p* < 0.01, and ^####^ *p* < 0.0001.

Hydrogen peroxide is only one of the components of ROS; we irradiated skin cells with UVA to produce excess ROS and induce damage. The cell viability of the group irradiated with UVA only was significantly reduced to 48%, whereas the mixture group showed a result of 86%, indicating a significant resistance to the decline in cell viability caused by UVA (Figure 5a). LDH and MDA were increased after UVA irradiation. This suggests that UVA promotes both death and lipid peroxidation; however, it was significantly inhibited by the hemin–tyrosine mixture (Figure 5b,c). In addition, the mixture-treatment group showed significantly improved cell viability and downregulation of LDH release and MDA accumulation, in contrast to the hemin-alone treatment group and the tyrosine-alone treatment group, revealing the synergistic protective role of tyrosine and hemin (Figure 5a–c).

UVA irradiation caused 29% of the cells to accumulate in the G2–M phase, whereas in the mixture-treated group, it accounted for merely 6% (Figure 5d). The apoptosis results showed that as few as 22% of the cells survived after UVA irradiation, and the remaining underwent apoptosis, whereas the survival rate of the cells treated with the mixture of hemin and tyrosine reached up to 67% (Figure 5e). Furthermore, both the cell cycle and apoptosis showed that the mixture of hemin and tyrosine was significantly more protective than hemin or tyrosine alone. These results indicate that the mixture significantly protected cells from UVA-induced apoptosis.

## 4. Discussion

Hemin catalyzes the reaction of tyrosine with hydrogen peroxide, and the catalytic mechanism is probably as follows:

(4) $$H_{2}O_{2}+Fe^{3+}\to Fe^{4+}=O$$

(5) $$Fe^{4+}=O+Tyr\to \;Fe^{4+}=O+Tyr$$

(6) $$Fe^{4+}=O+Tyr\to \;Fe^{3+}+Tyr+H_{2}O$$

(7) 2Tyr→ Tyr−Tyr

In this reaction, hydrogen peroxide, the electron donor, can oxidize the hemin (*Fe*^3+^) to the ferryl hemin radical (*Fe^*4*+^=O·*) (Equation (4)); then, this is converted into another form, ferryl hemin (*Fe^*4*+^=O*), through a reaction with tyrosine (Equation (5)). Additionally, tyrosine acts as an electron acceptor, reducing ferryl hemin (*Fe^*4*+^=O*) to hemin (*Fe^*3*+^*) and forming a tyrosine radical (Equation (6)). Finally, two tyrosine radicals can react rapidly to produce a dityrosine with fluorescent properties (Equation (7)) [25,26]. Briefly, the hydrogen peroxide in the system (hemin–H_2_O_2_–Tyr) is depleted through a catalytic reaction that uses tyrosine as a substrate, and significant amounts of dityrosine are produced. Description of the Michaelis constant for the catalyst is necessary; however, in the case of hemin oxidation, it is difficult to obtain the exact Michaelis constant. In fact, when hydrogen peroxide reacts with hemin, hemin is oxidized to ferryl hemin (*Fe^*4*+^=O·*) (Equation (4)), and this process is accompanied by the release of iron atoms and the degradation of hemin [23,27].

Physiologically, both hydrogen peroxide and hemin are present at traceable levels, but the concentration of tyrosine used in the present study was higher than the concentrations of the other two substrates in order to ensure the complete depletion of hydrogen peroxide and exert an antioxidant effect.

Tyrosine, a phenolic amino acid, can provide an electron or hydrogen atom for the free radical, and hemin’s scavenging effect also probably originates from the redox reaction with free radicals [28]. For the scavenging of hydroxyl free radicals, the Fenton reaction is inhibited due to the hydrogen peroxide being consumed by the mixture of hemin and tyrosine, thereby reducing hydroxyl free radicals. The synergistic effect of hemin and tyrosine in scavenging free radicals may be attributed to the fact that these free radicals produce hydrogen peroxide in the degradation process, leading to a certain catalytic effect. Additionally, the scavenging activity of hemin for DPPH· was inferior to that of the positive control at 0–0.45 mM (Figure 3c). A possible explanation is the unique porphyrin structure of hemin, which results in a poor electron-accepting activity for DPPH·.

We demonstrated a protective effect of the mixture (hemin–Tyr) on the oxidative damage of cells induced by UVA. Mitochondrial membrane permeability is altered by ROS, triggering apoptosis [29]. The antiapoptotic effect may be attributed to the scavenging of ROS, which protected the mitochondria from ROS attack and improved cell viability.

However, hemin and tyrosine can absorb ultraviolet rays like sunscreens, which may also contribute to the protective effect of cells. On the other hand, nuclear factor erythroid-2-related factor 2 (Nrf2) is responsible for the cellular response to oxidative stress. When it is activated by hemin, the expression of antioxidant enzymes in cells increases, and those can attenuate the oxidative stress caused by GOX or ultraviolet rays [30,31,32].

Both hemin and tyrosine exhibited inherent metabolic mechanisms and processes in vivo, showing potential as protective agents for combating UVA-induced skin damage. However, skin is a multilayered, complex organ that consists of many cells, including fibroblasts, melanocytes, etc. Under excessive ROS, these cells promote more complex physiological phenomena, such as collagen degradation, pigmentation, and wrinkle generation, eventually leading to skin aging and melasma [33,34,35]. Given the above reasons, the mixture of hemin and tyrosine may also have a protective effect against UVA-induced damage to other skin cells and provide more comprehensive skin protection. Excessive ROS induce oxidative stress apart from that involved in skin damage, as seen in ischemia–reperfusion injury and neurodegenerative diseases [36,37]. Hence, the mixture of hemin and tyrosine may have therapeutic effects in these conditions. However, the solubility of tyrosine was limited, requiring further efforts to find a more suitable catalytic substrate than tyrosine or to chemically modify tyrosine to increase its solubility for subsequent in vivo studies.

## 5. Conclusions

This research shows that hemin can catalyze the reaction between hydrogen peroxide and tyrosine. Moreover, the mixture of hemin and tyrosine has synergistic free-radical-scavenging properties and can resist the oxidative damage caused by UVA. This research highlights a new strategy for developing agents for protection against UVA.

## Data Availability

Not applicable.
